# A study of the moving rate of positive results for use in a patient‐based real‐time quality control program on a procalcitonin point‐of‐care testing analyzer

**DOI:** 10.1002/jcla.24320

**Published:** 2022-03-07

**Authors:** Yili He, Daqing Gu, Xiangzhi Kong, Zhiqiang Feng, Weishang Lin, Yunfeng Cai

**Affiliations:** ^1^ Clinical Laboratory The Fifth People's Hospital of Panyu Guangzhou China

**Keywords:** internal quality control, moving rate of positive results, point‐of‐care testing, procalcitonin

## Abstract

**Objective:**

To establish an applicable and highly sensitive patient‐based real‐time quality control (PBRTQC) program based on a data model constructed with patients’ results of a procalcitonin point‐of‐care testing (POCT) analyzer.

**Methods:**

Patients’ results were retrospectively collected within one year. The Excel software was used to establish quality control (QC) programs of the moving average (MA) and the moving rate of positive results (MR). A Monte Carlo simulation was used to introduce positive and negative biases between 0.01 and 1 ng/ml at random points of the testing data set. Different parameters were used to detect the biases, and the detection efficiency was expressed using the median number of patient samples affected until error detection (MNPed). After comparing the MNPeds of different programs, MA and MR programs with appropriate parameters were selected, and validation plots were generated using MNPeds and maximum number of the patient samples affected (MAX). *β* curves were generated using the power function of the programs, the performances were compared with that of the conventional QC program.

**Results:**

Neither the conventional QC nor MA program was sensitive to small bias, While MR program can detect the minimum positive bias of 0.06 ng/ml and negative of 0.4 ng/ml at an average daily run size of 10 specimens, with FRs < 1.0%, *β*s < 1%.

**Conclusion:**

The MR program, which is more sensitive to small biases than conventional QC and MA programs, with low FR and *β*. As such, it can be used as a PBRTQC program with high performance.

## Graphical abstract

Collecting patients’ results within one year to establish PBRTQC programs of the MA and the MR. The parameter of false alarm rate (FR) 1% was eliminated. The Monte Carlo simulation within VBA was used to introduce ± 0.01 ~ 1 ng/ml at random points of the testing set. Different parameters within a specific interval were used to detect the biases, and the detection efficiency was expressed using the MNPed. The parameters of the minimum MNPeds were selected as the optimal parameters of MA and MR programs, and validation plots were generated using MNPeds and MAX of the patient samples affected. *β* curves were generated simultaneously, and the sensitivities were compared with that of the conventional internal quality control program. The ability of conventional QC to detect a 0.05 ng/ml bias using the 13S/22S/R4S multi‐rule.

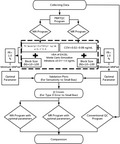

## 1 INTRODUCTION

Point‐of‐care testing (POCT) is currently a means for clinical laboratories to provide test results. However, with the rapid development of new methods and technologies, clinicians are paying more attention to the advantages and disadvantages of POCT.^1^ Traditional quality control (QC) programs analyze only QC products of two concentrations at a time,^2^ which may not be sufficient for the rapid detection of analytical errors affecting patient outcomes.^3^, ^4^ Moreover, artificial serum can produce matrix effects.^5^ Although some clinicians use patient samples as internal QC products,^6^ the precision of the sample concentration and the extremely high requirements for laboratory storage render this approach infeasible in most primary laboratories. For this reason, a patient‐based real‐time quality control (PBRTQC) method has been proposed for the QC analysis of population data by calculating the mean, median, or standard deviation of real‐time results.^7^, ^8^ Among the PBRTQC methods, the moving average (MA) program is the most widely used. However, the MA program cannot rapidly detect all types of biases such as small biases.^9^

As one of the early clinical diagnostic indicators of infection, procalcitonin (PCT) is a highly specific and sensitive biomarker.^10^ However, it associates with systematic errors due to factors such as instrument failure, poor operator habits, or changes in reagents and environment, all of which can affect clinical judgment. The sensitivity of the QC program determines whether small system errors can be rapidly detected so that appropriate measures can be quickly implemented. Liu et al.^11^ demonstrated that the moving sum of the number of positive patient results for prostate‐specific antigen, as the QC procedure, can rapidly detect a positive bias of 0.03 mg/L, which is impossible with conventional QC and MA programs. However, in previously published studies,^12^, ^13^, ^14^ the project clinical decision point was taken as the parameter of the PBRTQC program. In this study, we identify optimal parameters to detect small errors for the programs through simulations based on the data model, strive to shorten the time required for the QC programs to detect systematic errors under the conditions that both type I and II errors are within acceptable limits, and ensure the clinical accuracy of the POCT programs by monitoring the quality of PCT detection.

## 2 MATERIALS AND METHODS

### 2.1 General information

The results of 2434 PCT samples tested in a laboratory at the Fifth People's Hospital of Panyu District, Guangzhou from July 2019 to June 2020 were retrospectively collected. The results of 20 proficiency samples and 146 internal QC (IQC) samples were excluded. The rule of 4_1S_/1_3S_/2_2S_ was followed by the laboratory, and no out‐of‐control points caused by PCT analyzers, methods, or reagents were observed in the IQC chart during this period. The PCT results were verified by SPSS 22 software (IBM, Armonk, NY, USA), and the data showed a skewed distribution (*p* < 0.05). Data simulation analysis was performed by Excel 2007 software (Microsoft, Redmond, WA, USA). After considering the small specimen size on weekends and holidays, the daily run size for weekdays was set to approximately 10 specimens/day.

### 2.2 Instruments and reagents

The TZ‐301 analyzer (ReLIA, Shenzhen, China), the PCT detection kit (ReLIA) with a minimum detection limit of 0.02 ng/ml, and IQC analytes (Acusera series, RANDOX, city, UK) were used in this study.

### 2.3 Efficacy of traditional IQC to detect biases

The Westgard 4_1S_/1_3S_/2_2S_ multi‐rule method was used weekly to analyze the IQC analytes of two concentrations. The mean values of the 12‐month QC results were 1.50 ng/ml and 19.88 ng/ml, respectively, and the analytical coefficients of variation (calculated as standard deviation/mean, SD/mean, CV) were 4.40% and 3.98%, respectively. We assumed that the IQC concentrations obeyed a normal distribution. Thus, to obtain the probability of a critical bias triggering a QC rule, we calculated the standard *z* value for the probability of a QC result greater than *χ* SD (*χ* = 1, 2, or 3) in presence of a critical bias as follows^12^:

$$z=\frac{\left({\text{Mean}}_{\text{old}}+\chi \times {\text{Mean}}_{\text{old}}\times \text{CV}\right)‐\left({\text{Mean}}_{\text{old}}+\text{critical}\;\text{bias}\right)}{{\text{Mean}}_{\text{new}}\times \text{CV}}$$

which could be expressed as:

(1) $$z=\frac{\chi \times {\text{Mean}}_{\text{old}}\times \text{CV}‐\text{critical}\;\text{bias}}{{\text{Mean}}_{\text{new}}\times \text{CV}}$$

where Mean_old_ and Mean_new_ are the averages of the QC concentrations before and after a bias was introduced, respectively. This could be expressed as follows: Mean_new_ = Mean_old_ + critical bias. For example, the *z* value for the probability that the 1_2S_ rule was triggered in the presence of a 0.05 ng/ml bias via a QC analyte with low concentration of 1.50 ng/ml was:

$$z=\frac{2\times 1.50\times 0.044‐0.05}{(1.50+0.05)\times 0.044}=1.202$$

After consulting the *z*‐table, we obtained *p* = 0.885, and the QC result greater than 2SD was (1‐*p*) ×100% = 11.5%, and the probability of two consecutive QC results greater than 2SD (i.e., 2_2S_ rule) was (1‐*p*)^2^ × 100% = 1.32%. Similarly, when *N* = 1, the *z* value was 0.2346, *p* = 0.593, and (1‐*p*) × 100% = 40.7%, so the probability of obtaining four consecutive QC results greater than 1SD (i.e., 4_1S_ rule) was (1‐*p*)^4^ × 100% = 2.75%.

### 2.4 Determination of MA program parameters

The MA program was set up in three parts: (1) the exclusion of values above or below a certain threshold by applying a truncation limit (TL), (2) the MA calculation method, which included the MA algorithm and block size (*N*), was defined as the number of patient results to be averaged in the algorithm; and (3) the control limit (CL). The TL was used to minimize the impact of the extreme results on the dataset, which could reduce the false rejection rate (FR).^15^ The MA program was expressed as follows:

(2) $$Z_{\left(\alpha \right)}=\frac{X_{\left(\alpha \right)}+X_{\left(\alpha ‐1\right)}+X_{\left(\alpha ‐2\right)}+\cdots +X_{\left(\alpha ‐n+1\right)}}{N}$$

where *Z*_(_ *_α_*_)_ is the calculated average value of the PCT result, and *X*_(_ *_α_*_)_ is the result of sample *α*. According to the time series data, the MA program continuously operated on a term‐by‐term basis and calculated the sequential average, including a certain block size. Each time a new result was merged into the block, the oldest result was discarded, and the average value of the block was recalculated for comparison with the predefined CL. The CLs were set using the mean and SD of the MA as follows:

(3) $$\text{Control}\;\text{limit}={\text{Mean}}_{\text{MA}}\pm 3\times {\text{SD}}_{\text{MA}}$$

### 2.5 Determination of MR program parameters

The MR program also consisted of three parts, but instead of using the TL to smooth the dataset, the MR program used a cut‐off value (COV) for the binary conversion of the data set. The returned state was “0” when the original value was ≤COV; otherwise, the returned state was “1.” The program was as follows:

(4) $${\text{MR}}_{\left(\alpha \right)}=\frac{T_{\left(\alpha \right)}+T_{\left(\alpha ‐1\right)}+\cdots +T_{\left(\alpha ‐n+1\right)}}{N}\times 100\%$$

where MR_(_ *_α_*_)_ is the operating moving rate within a block, and *T*_(_ *_α_*_)_ is the binary conversion value (1 or 0) of the PCT concentration of the sample *α*. New MR values were obtained for each new PCT test result in successive MR operations. The CLs were set using the mean and SD of the MR as follows:

(5) $$\text{Control}\;\text{limit}={\text{Mean}}_{\text{MR}}\pm 3\times SD_{\text{MR}}$$

The efficacy of the bias detection under MA and MR programs with different TLs/COVs and *N* was expressed as the median number of patient samples affected until error detection (MNPed).

### 2.6 Simulation and validation of bias detection in MA or MR

To investigate the performance of MA and MR programs under different parameters, the first 1000 data files served as the training set. The mean and SD of MR/MA were calculated from this, and the remaining 1434 data files served as the testing set. CLs were set according to Equations (3) and (5). The FRs of MA and MR programs were computed at the same time. The visual basic for applications (VBA) development tool within Excel software was used to program the Monte Carlo simulation to introduce continuous positive or negative biases between 0.01 and 1.0 ng/ml at 100 random positions of the testing data set, respectively. If the result was <0.02 ng/ml, then the calculated value was 0.02 ng/ml. The MNPeds and maximum number of patient samples affected until error detection (Max) with different parameter combinations were calculated. After comparing the MNPeds of MA and MR programs with different Ns, the MA and MR programs with optimal parameters were selected for validation, and the type II error *β* value was calculated for each round of validation. Bias detection and validation curves were then plotted according to the description, with the x‐axis representing the bias introduced and the y‐axis representing the number of patient samples affected. MNPeds were plotted as bar graphs, and MAX values were plotted as error lines. Small values of MNPed, FR, and *β* were expected for the PBRTQC program, and ideally, errors were detected in the daily run size, that is, MAX error lines did not exceed the daily run size.

## 3 RESULTS

### 3.1 Efficacy of conventional QC procedures to detect biases

The probability of triggering different QC programs by introducing biases between 0.01 and 1.0 was calculated, and data are shown in Table 1. The results indicated that a bias of 0.24 ng/ml or more was required to trigger the 4_1S_ rule first, with 95.5% probability, while the probability of triggering the same rule with a 19.88 ng/ml QC product was even lower.

**TABLE 1:** Probability of detection of different biases by the conventional multi‐rule internal quality control program

	Bias magnitude (ng/ml)						
	QC Rule	0.01	0.05	0.1	0.24	0.5	1.0
Level 1 QC 1.50 ng/ml	1_3S_	0.23%	1.5%	8.2%	70.8%	100%	100%
	2_2S_	0.11%	1.3%	11%	84.8%	100%	100%
	4_1S_	0.16%	2.8%	22%	95.5%	100%	100%
Level 2 QC 19.88 ng/ml	1_3S_	0.14%	0.17%	0.21%	0.39%	1.0%	4.9%
	2_2S_	0.055%	0.071%	0.097%	0.22%	0.83%	5.8%
	4_1S_	0.070%	0.094%	0.14%	0.34%	1.7%	13%

Note

The conventional QC program uses 1.50 ng/ml quality control product to trigger the 4_1_S rule first, which requires a bias of more than 0.24 ng/ml, with 95.5% probability.

Abbreviation: QC, quality control.

### 3.2 Determination of MA and MR program parameters

As the programs monitored the layout of the data sequence rather than the patient's clinical background,^16^ instead of using the clinical decision points, in the MA program, we expanded the selection range of the TL to an interval, that is, the population mean ± *i* × SD, and measured the most sensitive parameters within the selected interval. In addition, due to the large overall patient SD (4.21 ng/ml), a smaller *i* value was required to converge the discrete degree. When *i* = 0.1, the data rejection rate was 85.57%, which was too high, rendering the monitoring program ineffective. When *i* = 0.2–0.8, the data rejection rate was 6.0%–2.59%, which rejected approximately 5% of the extreme values and maximized the utilization of the patient data. In the MR program, we first set the random block size to *N* = 50 and introduced a target bias of 0.05 ng/ml. Pre‐analysis found that minimum MNPeds (22–46) appeared in an interval of COV = 0.03–0.07 ng/ml, and that the MNPeds increased with the increase of the COV; therefore, the COV was set to 0.02–0.08 ng/ml before simulated in detail. The above programs used *N* = 10–100 as the block size to simulate the performance of the bias detection, and the CL and FR rates are shown in Table 2.

**TABLE 2:** Parameters of MA and MR program with various block sizes

MA procedure						MR procedure					
*i*	Truncated data	Block Size	LCL	UCL	FR (%)	COV	Positive Rate	Block Size	LCL	UCL	FR (%)
0.2	6.0%	10	<0.02	0.34	1.89	0.02	94.57%	10	78.37	>100	1.53
		20	<0.02	0.27	0.66			20	85.01	>100	10.81
		30	0.03	0.24	0.18			30	84.24	>100	4.28
		40	0.05	0.23	0.27			40	85.80	>100	6.31
		50	0.06	0.22	0.27			50	87.03	>100	7.26
		60	0.07	0.21	0.49			60	88.03	>100	6.15
		70	0.07	0.20	1.04			70	88.26	>100	10.07
		80	0.08	0.20	0.54			80	89.49	>100	7.69
		90	0.08	0.20	1.00			90	90.15	>100	11.05
		100	0.08	0.20	1.10			100	90.66	>100	8.74
0.3	4.8%	10	<0.02	0.38	2.56	0.03	81.46%	10	46.73	>100	0.37
		20	<0.02	0.31	3.48			20	58.73	>100	0.21
		30	0.02	0.28	1.79			30	63.28	>100	0.04
		40	0.04	0.26	1.36			40	66.29	>100	0.13
		50	0.05	0.25	0.62			50	69.24	98.15	0.50
		60	0.06	0.24	0.62			60	71.35	96.13	0.55
		70	0.07	0.23	0.85			70	72.70	94.89	0.97
		80	0.07	0.23	0.76			80	74.13	93.56	6.75
		90	0.07	0.22	0.85			90	75.29	92.46	7.51
		100	0.08	0.22	1.04			100	76.24	91.52	12.85
0.4	4.4%	10	<0.02	0.46	2.11	0.04	69.42%	10	25.48	>100	0.21
		20	<0.02	0.37	1.30			20	38.63	>100	0.33
		30	<0.02	0.34	1.13			30	44.79	95.47	0
		40	0.02	0.32	1.09			40	48.69	91.83	0.08
		50	0.02	0.31	0.66			50	51.84	88.79	0.08
		60	0.03	0.29	0.35			60	54.57	86.15	0.63
		70	0.04	0.28	0.75			70	56.57	84.22	0.93
		80	0.04	0.28	0.53			80	58.10	82.79	1.06
		90	0.05	0.27	0.49			90	56.31	81.61	0.90
		100	0.05	0.27	0.76			100	60.04	80.81	0.73
0.5	3.8%	10	<0.02	0.52	2.36	0.05	60.58%	10	12.94	107.39	0.08
		20	<0.02	0.42	1.81			20	26.04	94.31	0
		30	<0.02	0.38	1.38			30	32.01	88.41	0
		40	<0.02	0.36	1.04			40	36.28	84.34	0
		50	0.01	0.34	0.57			50	40.19	80.59	0.13
		60	0.02	0.33	0			60	43.26	77.60	0.42
		70	0.02	0.32	0.22			70	45.39	75.56	1.23
		80	0.03	0.32	0.40			80	46,71	74.33	0.98
		90	0.03	0.32	0.93			90	47.54	73.53	0.77
		100	0.03	0.31	0.53			100	48.13	72.92	0.73
0.6	3.1%	10	<0.02	0.61	1.79	0.06	53.84%	10	3.30	>100	0
		20	<0.02	0.48	0.51			20	16.80	88.77	0.04
		30	<0.02	0.43	0.21			30	22.56	84.11	0.08
		40	<0.02	0.39	0.30			40	26.89	79.96	0
		50	<0.02	0.37	0			50	30.69	76.33	0
		60	0.03	0.36	0			60	33.96	73.15	0.08
		70	0.04	0.34	0.17			70	36.20	70.98	0.17
		80	0.05	0.34	0.13			80	37.71	69.55	0.34
		90	0.05	0.33	0.09			90	38.46	68.81	0.68
		100	0.06	0.33	0.04			100	38.94	68.28	0.30
0.7	2.91%	10	<0.02	0.61	2.68	0.07	48.62%	10	<0	97.41	0.17
		20	<0.02	0.48	0.60			20	11.28	84.91	0.04
		30	<0.02	0.43	0.21			30	17.18	79.01	0
		40	<0.02	0.39	0.30			40	21.81	74.51	0
		50	0.03	0.37	0.09			50	25.74	70.73	0
		60	0.03	0.36	0.35			60	28.82	67.74	0
		70	0.04	0.34	0.17			70	30.74	65.86	0.04
		80	0.05	0.34	0.18			80	31.76	64.90	0.34
		90	0.05	0.33	0.22			90	32.21	64.46	0
		100	0.06	0.33	0.04			100	32.43	64.19	0
0.8	2.59%	10	<0.02	0.69	1.82	0.08	43.86%	10	<0	88.42	0.74
		20	<0.02	0.54	0.38			20	9.38	75.35	0.21
		30	<0.02	0.47	0.38			30	14.57	70.14	0.17
		40	<0.02	0.44	0.26			40	18.38	66.43	0.33
		50	0.03	0.42	0.09			50	21.45	63.51	0.08
		60	0.02	0.40	0.09			60	23.73	61.32	0.34
		70	0.03	0.39	0.13			70	25.12	59.94	0.72
		80	0.04	0.38	0.13			80	25.90	59.17	0.42
		90	0.05	0.38	0.09			90	26.30	58.74	0.60
		100	0.05	0.37	0			100	26.61	58.33	0.13

Note

The *i* was used in the MA procedure to eliminate a certain number of outliers, for example, *i* = 0.2 means that the population mean ± 0.2 SD was the truncated limit, this eliminated data accounting for 6% of the population data. When *N* = 30, the lower and upper control limits were 0.03 and 0.24, respectively, and the false rejection rate was 0.18%. The cut‐off value was used in the MR method to assign binary values to patient data, for example, COV = 0.03, if the patient result was greater than the COV, then it was regarded as “1”; otherwise, it was regarded as “0.” When *N* = 10, the lower control limit was 46.73%, and the upper control limit was invalid. The false rejection rate was 0.37%.

Abbreviations: COV, cut‐off value; FR, false rejection rate; LCL, lower control limit; MA, moving average; MR, moving rate; UCL, upper control limit.

### 3.3 MA or MR bias detection simulation

**FIGURE 1:**
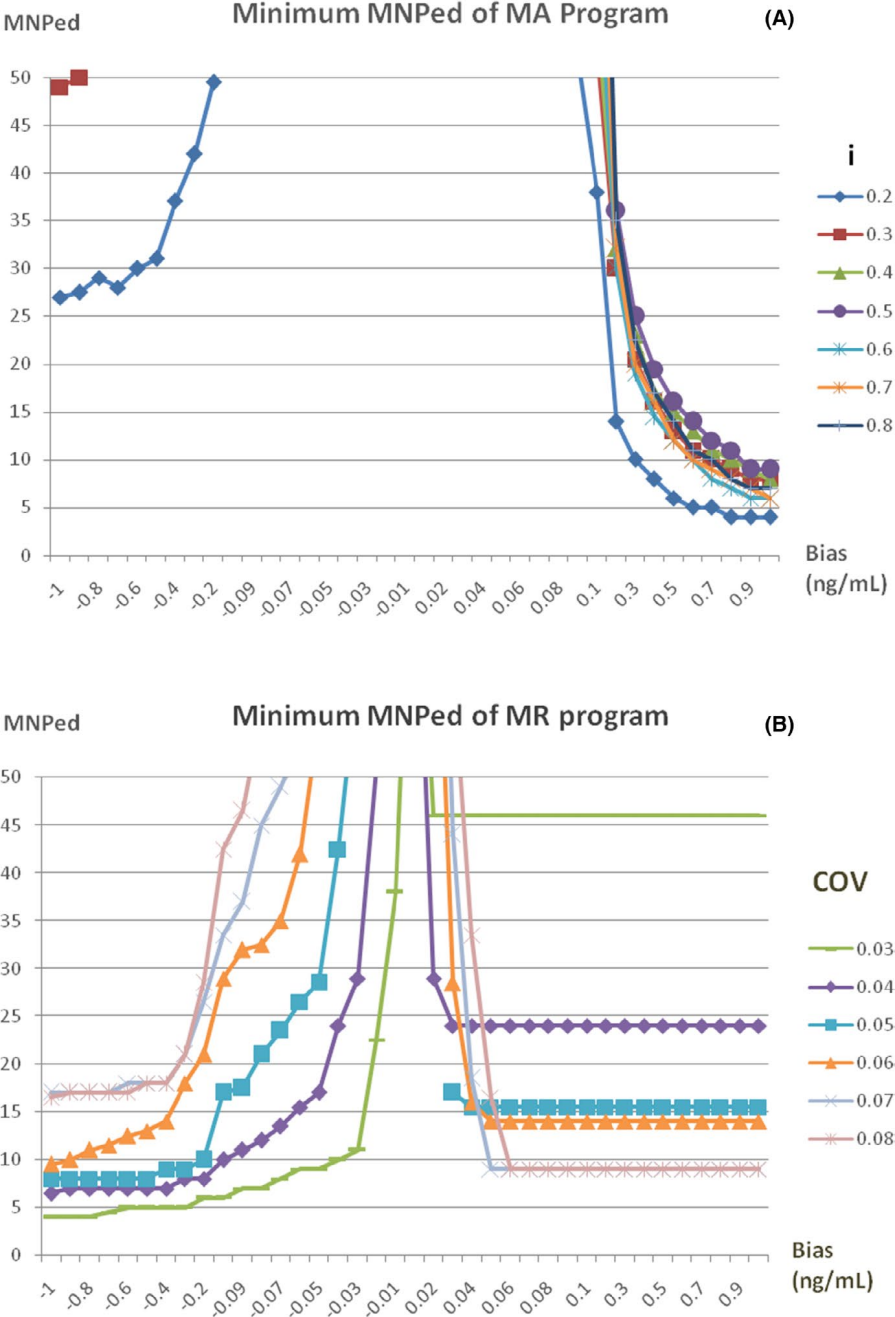
The minimum MNPed of different parameters and biases in MA and MR programs. (A) The minimum MNPed curves obtained by the MA program after simulating different COVs combined with different Ns to detect different errors and eliminating the parameters of FR ≧1.0%. For example, the curve of *i* = 0.3 is curve of the minimum MNPed obtained when running *N* = 50~90 with FR < 1.0%. Since the minimum MNPed value was concentrated on the curve with *N* = 0.2, *N* = 0.2 was selected. (B) is similar to the above, COV = 0.03 and COV = 0.07 were selected as parameters of MR program

The minimum of the MNPeds for MA and MR programs with different parameters are shown in Figure 1, parameters with a FR greater than 1% were eliminated. For the MA program, the minimum MNPed was when *i* = 0.2 (Figure 1A). The minimum MNPeds for negative and positive biases detected by the MR program were identified when COV = 0.03 and COV = 0.07, respectively (Figure 1B). Therefore, *i* = 0.2, as well as COV = 0.03 and 0.07, were selected to generate bias detection curves for MA and MR programs, respectively.

**FIGURE 2:**
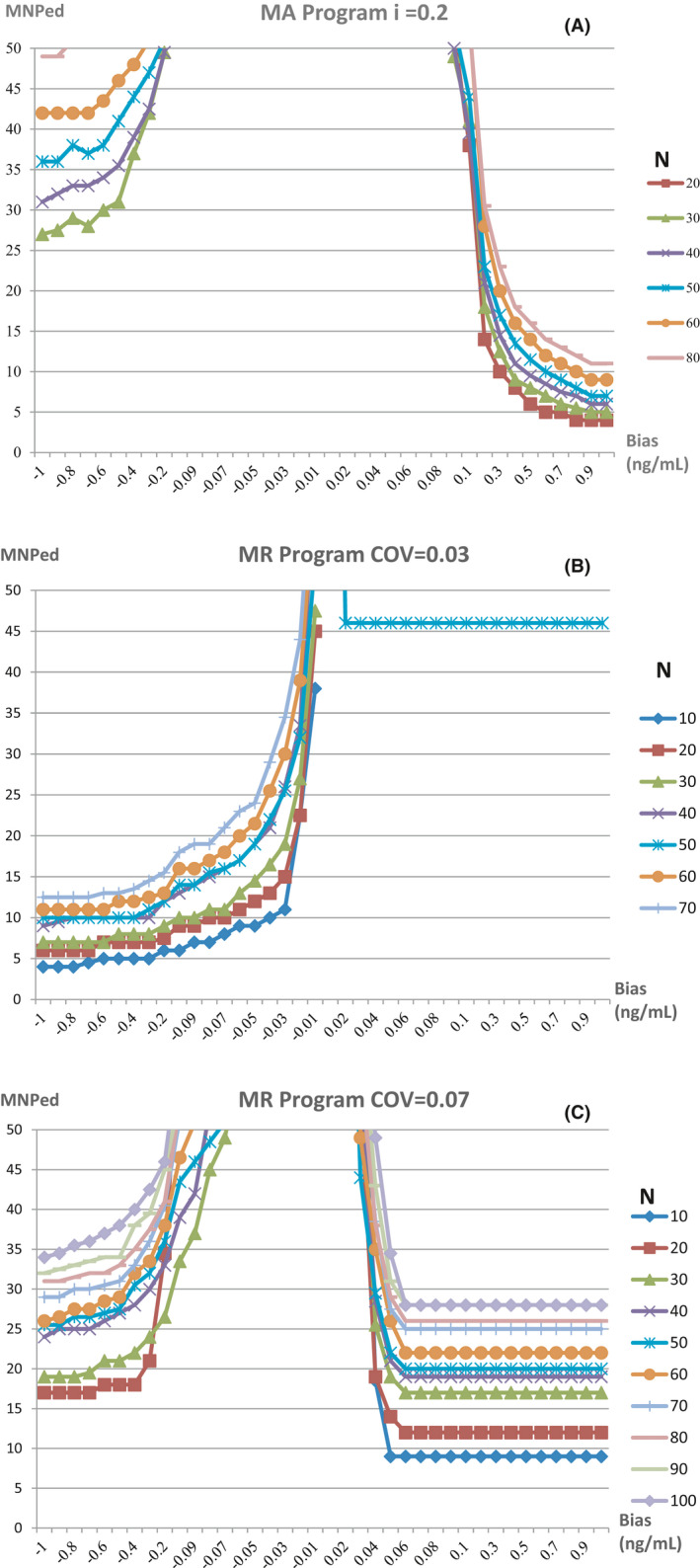
Bias detection curves for MA and MR programs with different block sizes. (A) MA program with *i* = 0.2. (B) MR program with COV = 0.03. (C) MR program with COV = 0.07. Different curves represent MNPeds measured by QC programs with different block sizes (*N*)

The bias detection curves for the two programs with different *N* after the TL or COV was determined are shown in Figure 2. For both programs, a larger *N* identified more positive and negative biases that could be simultaneously covered by the detection capability, although the detection sensitivity was reduced. The MA program was more sensitive to positive biases, whereas the MR program differed in sensitivity to positive or negative biases depending on the COV selected. As such, *i* = 0.2 and *N* = 20, as well as *i* = 0.2 and *N* = 30, were selected as the optimal parameters of the MA program, whereas COV = 0.03 and *N* = 10, as well as COV = 0.07 and *N* = 10, were selected as the optimal parameters of the MR program to generate the validation graphs for program detection.

### 3.4 Validation of MA and MR programs

**FIGURE 3:**
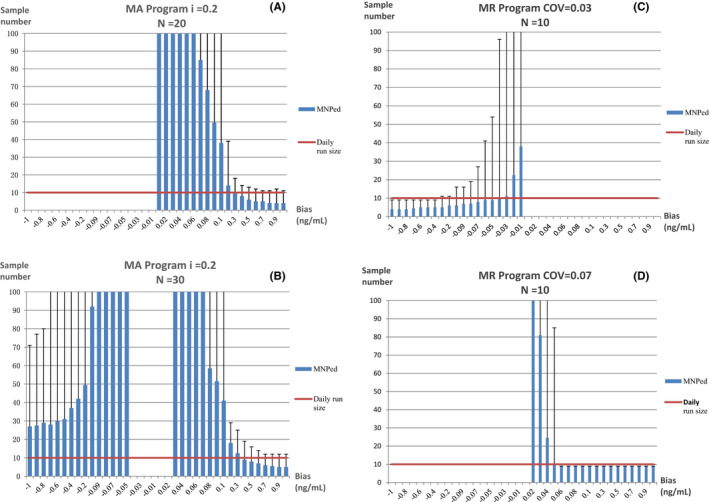
Validation charts for optimal MA or MR program for the bias detection. (A) MA program with *i* = 0.2 and *N* = 20. (B) MA program with *i* = 0.2 and *N* = 30. (C) MR program with COV = 0.03 and *N* = 10. (D) MR program with COV = 0.07 and *N* = 10. The blue strips represent MNPeds, the error lines represent the maximum number of results required for bias detection, and the red lines represent the daily run size of specimens

**FIGURE 4:**
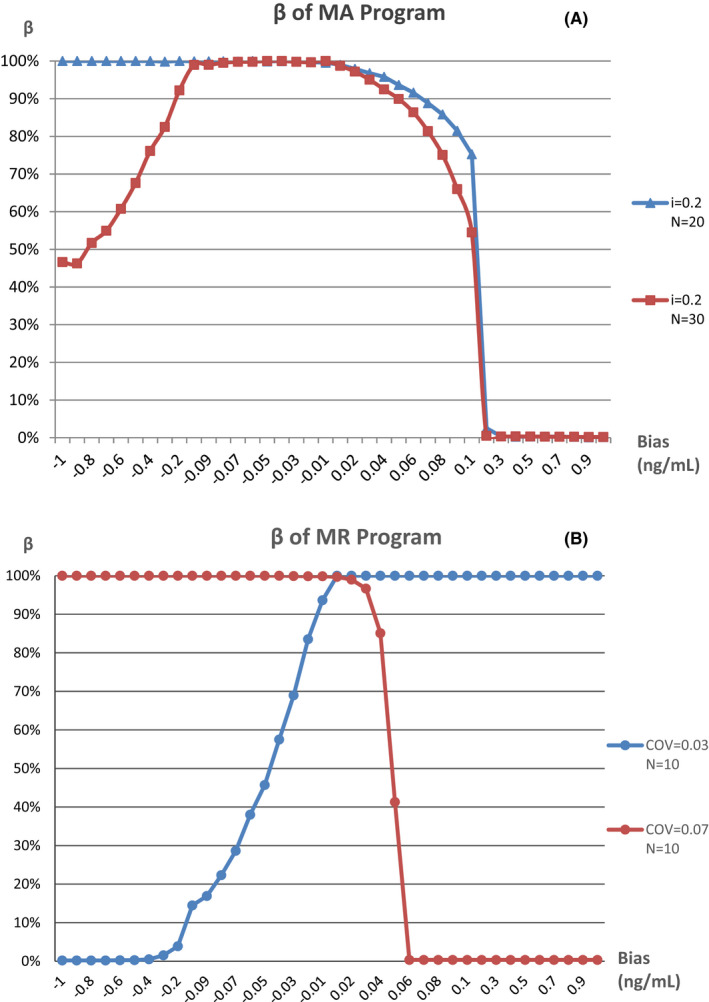
*β* Curves of bias detection for optimal MA and MR programs. *β* represents the false negative rate detected by the program after introducing bias, also known as type II error. (A), (B) The MA programs with *i* = 0.2, *N* = 20, and *i* = 0.2, *N* = 30, when bias ≥ 0.2 ng/ml, *β* < 1%. (C) The MR program with COV = 0.03, *N* = 10, when negative bias ≥ 0.4 ng/ml, *β* < 1%. (D) The MR program with COV = 0.07, *N* = 10, when bias ≥ 0.06 ng/ml, *β* < 1%

The MA and MR validation plots drawn with the optimal parameters are shown in Figure 3, The MR program is more sensitive to small biases, with a positive bias of 0.06 ng/ml or above and a negative bias of 0.4 ng/ml or above detected at a rate of 100% in a day, but only positive or negative biases could be detected by the MR program with the two different combinations of parameters, separately. On the contrary, The MA program is not sensitive to small biases, MNPeds of which are significantly greater than MR. The curves of *β* value, which is the rate of false negative that occurred in QC programs when bias was introduced, for each program are shown in Figure 4. The curves showed that when the MR program detects a positive bias of ≧0.06 ng/ml, *β* = 0.33%, or negative bias ≧0.4 ng/ml, *β* ≦ 0.50%, as such, while when the ma program detects positive bias ≧0.2 ng/ml, *β* < 1.0%, these type II errors indicating that the performance of MR Program detecting small biases was more stable than the MA program.

**FIGURE 5:**
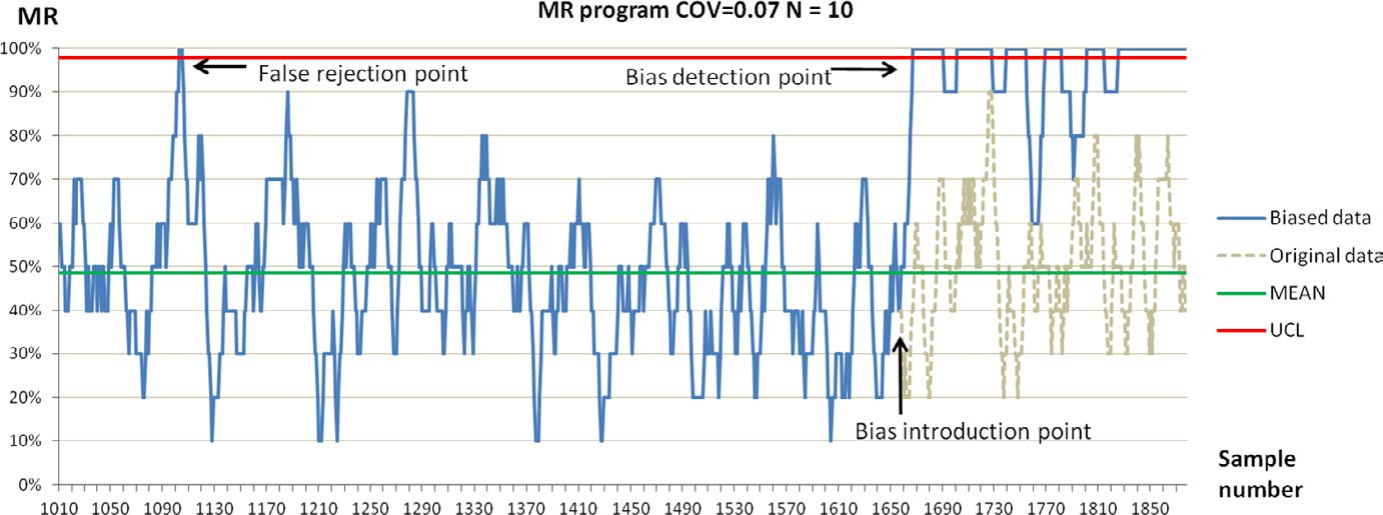
Simulation diagram of the MR program to detect systematic errors. The green horizontal line represents the average MR of the positive results. The red horizontal line indicates the upper control limit. The dashed lines represent the original data sets. The blue line represents the data set after introducing bias to the simulation at a random point. For the MR program with COV = 0.07 and *N* = 10, the lower limit of the control was < 0%, so there was no lower control limit in the sample diagram

The QC plot shows how the MR program detected systematic errors. The difference between an FR signal and true bias during the detection of the PCT concentration by introducing biases at random positions is shown in Figure 5.

## 4 DISCUSSION

PCT has high clinical value as an early indicator in the diagnosis of systemic bacterial infections, especially sepsis, with a reference value of < 0.05 ng/ml in healthy individuals and 0.05 ng/ml in those with mild systemic inflammatory responses.^17^ However, based on our stimulation, conventional QC, with a 95% daily probability of triggering the 4_1S_ rule that required a bias of 0.24 ng/ml or more, was not clinically sensitive, which may alter clinical interpretation and lead to incorrect treatment. And a PBRTQC model was established with the experimental data of patients within one year to run the MA and MR program, and the results suggested that MA program could not detect positive and negative bias of 0.01–1.0 at an average daily run size of 10, this indicating that neither the conventional QC nor MA program was suitable for detecting small systematic biases, which is consistent with the reported results.^18^ The MR program with simulated optimal parameters could consistently detect positive of 0.06 ng/ml and above, or negative of 0.4 ng/ml and above biases, at an average daily run size of 10. A comparison of the two PBRTQC programs revealed that the MA program rejected as many outliers as possible to narrow the CL and to improve the detection sensitivity, but the increased rejection rate of the data reduced the frequency of calculating patient results, leading to possible delays in rejection and the possibility of not detecting particularly large biases.^19^, ^20^ After converting the results into the binary state, the MR program had no TLs and excessive concentrations for judgment, but had higher data utilization than the MA program, so it is very suitable for analyses with small volumes of data such as that in this study. Furthermore, the PCT results of the population showed a skewed distribution, leading to a reduction in the applicability of the MA method.^21^ On the one hand, if the moving median method is used, then it is more difficult to interpret the results^22^; on the other hand, it is difficult to estimate the standard deviation of the median, and the mathematical relationship between it and the mean standard deviation complicates the program.^23^ Therefore, the MR method with relatively simple operations is more applicable to similar distribution models.

Contrary to the previous studies, this study was based on the data model itself, and the parameter interval with the highest sensitivity to the target error (0.05 ng/ml) was first estimated by pre‐simulation. Detailed simulation was subsequently performed to determine the optimal parameter combination, which was free from the limitation of only selecting specific clinical decision points as the parameters. Although the number of simulations increased, this method provides the possibility of identifying more sensitive parameter combinations. In addition, a small block size (*N* ≤ 100) was used, and the smallest block size (*N* = 10) was found to have the lowest MNPed in most cases, but it leads to a possible high FR, simultaneously. Furthermore, it was found that the MR program could select double COVs as the parameters to detect positive or negative biases, which may have been related to the distribution frequency on both sides of the peak value of the data model. The MR parameters can be different for different data distributions, and the applicability of the double COVs to other data models requires further experimental validation. However, similar to the multi‐rule Westgard program with different performance, our approach is acceptable if it can meet the needs of a robust QC system. In summary, the MR program with appropriate parameters is highly sensitive to small systematic biases. With the FR rate and type II errors within acceptable limits, as well as simple calculation procedures for implementation at no additional cost, the MR program can automatically analyze data entered into the form. The QC program with multiple parameters can also be selected to ensure the sensitivity to biases in different directions as a supplement to or even outperform the existing conventional QC program.^24^

This study could not exclude the possibility of not detecting system errors in the dataset, and the original data were obtained after instrument maintenance and calibration to ensure the reliability of the results. Additionally, the biases in the experimental process may have been more complex and variable. However, we used VBA development tools to perform simulation operations for system biases of different directions and sizes, so as to ensure the credibility of the results. Moreover, by analyzing the sensitivity of the MR program and the rule of selecting the parameters, although the sensitivity of detecting biases in different directions is not equal, the high sensitivity of the MR program was demonstrated for small biases, which compensates for the deficiency of the MA method. The daily data size of the POCT analyzer in this study was relatively small, and it is precisely for this reason that efficient QC programs are needed to shorten the time to detect errors, to give full play to the effectiveness of the PBRTQC method while allowing for a range of errors, and to save laboratory costs. After all, there are likely to be experiments with a low volume of specimens in every laboratory.

According to the data of the Center for Medicine and Medicaid Services, 32% of POCT operators in the United States do not perform QC, and 20% of operators do not operate QC analytes in a standard manner.^25^ The *GB*/*T 29790*–*2020 Point*‐*of*‐*Care Testing (POCT)*‐*Requirements for Quality and Competence*, which was published in China, establishes the requirements for the quality assurance capability of POCT products, but there are no specific provisions for the practices of POCT operators. Therefore, there are many issues that need to be resolved, and technicians need to develop more sensitive and intelligent QC programs to address issues such as requiring additional manual operation steps when processing data, failing to judge true or false rejection signals, and traceability when result is outside the control limits. As such, expected results would be obtained during the QC process and be continuously improved, thereby allowing laboratory personnel to focus more on solving clinical problems.

## CONFLICT OF INTEREST

The authors confirm that they have no conflicts of interest.

## AUTHOR CONTRIBUTIONS

Yunfeng Cai designed the research, analyzed the data and wrote the manuscript. Yili He performed the research, analyzed the data and wrote the manuscript. Daqing Gu, Xiangzhi Kong, Zhiqiang Feng, Weishang Lin contributed to preparing the experimental reagents, sample detection and data collection.

## Data Availability

All data and material used for the manuscript is available from the corresponding author.
